# A Comparison of the Structural Changes and IgG Immunobinding Activity of Parvalbumin in Salangid Icefish (*Neosalanx taihuensis*) After Glycation and Ultra-High Pressure Treatment

**DOI:** 10.3390/foods14050856

**Published:** 2025-03-02

**Authors:** Ying Huang, Yang Hu, Jiawei Liu, Haiying Liu

**Affiliations:** School of Food Science and Technology, Jiangnan University, Wuxi 214122, China; 6220111044@stu.jiangnan.edu.cn (Y.H.); 6230112026@stu.jiangnan.edu.cn (Y.H.); 6210112050@stu.jiangnan.edu.cn (J.L.)

**Keywords:** *Neosalanx taihuensis*, parvalbumin, antigen specificity, ultra-high pressure treatment, glycation

## Abstract

The aim of this study was to compare the effects of glycation and ultra-high pressure (UHP) treatment on the structure and IgG immunobinding activity of Salangidae icefish PV. The Circular Dichroism (CD) and Fluorescence Spectroscopy (FS) findings indicated that the glycation significantly affected both the secondary and tertiary structures of PV. However, the impact of UHP processing on the structure of PV was found to be less significant compared to the glycation. Western Blot analysis also revealed that the glycation markedly reduced the antigen specificity of PV. Conversely, UHP treatments at 300 MPa and 400 MPa slightly decreased the antigen specificity, whereas lower or excessively high pressures did not have a substantial impact. This research contributes valuable insights into strategies for reducing the allergenic potential of Salangid icefish.

## 1. Introduction

Allergic diseases are abnormal immune responses caused by allergens and are common conditions in humans [1]. Currently, allergic diseases have become the sixth most common chronic disease worldwide [2]. Fish and other aquatic products are one of the major sources of food allergens [3]. In the United States, the prevalence of allergy to fin fish varied with age, spanning from 0.6% in children to a peak of 0.9% among adults [4,5]. In Canada, the incidence of fish allergy was approximately 0.5% [6]. Among school-aged children, the average sensitization rates to any type of fish ranged from 0.2% in Germany to 1.3% in Iceland [7]. In Asia, the highest prevalence of self-reported fish allergy was in the Philippines at 2.3% and Vietnam at 1.6% [8,9].

The principal allergens found in fish include parvalbumin (PV), aldolase, and endolase [10]. PV, an intracellular calcium-binding protein, plays a crucial role in the relaxation of fast-twitch muscle fibers [11,12]. PV is an acidic protein (pI: 4.5–5.1) that belongs to the EF-hand protein family and has an approximate molecular weight of 12 kDa [13]. Ef-hand proteins are proteins with EF-hand motif. This motif is a helix-loop-helix structure usually made up of a sequence of 12 residues [14,15].

Studies have shown that more than 90% of individuals with fish allergies exhibit allergic reactions to PV [16]. Typically, a single fish species contains several isoforms of PV, which may vary in stability and IgE reactivity [17]. Research on IgE cross-reactivity was based on conserved IgE epitopes present on PVs from different fish species [16,18,19].

Currently, common strategies for modifying food allergens to reduce their allergenic potential primarily involve glycation, phosphorylation, heating, ultra-high pressure treatment (UHP), ultrasound treatment, and enzymatic degradation [20,21,22]. The glycation, a non-enzymatic browning process, occurs when proteins react with reducing sugars. Glycation, the initial step in the Maillard reaction (MR), not only reduces the allergenicity of food allergens but also adds a detectable flavor to the food, making it a widely used technique in the food processing industry. UHP is a non-thermal processing technique that utilizes liquid (water or oil) as a pressure-transmitting medium to treat food materials under pressures ranging from 100 to 1000 MPa. Compared to traditional processing methods, UHP is gentler. UHP disrupts the structure of macromolecules by acting on non-covalent bonds, leading to the modification of macromolecular substances.

The Salangid icefish (*Neosalanx taihuensis*), a species belonging to the family Salangidae within the order Osmeriformes, was originally predominantly found in the Taihu region of the lower Yangtze River basin in China [23]. Currently, it is extensively cultivated in the pristine waters of southern China. The Salangid icefish is celebrated for its translucent flesh, rich in protein and calcium content, offering a delectable flavor and exceptional nutritional worth. Beyond its use in the culinary sector, Salangid icefish is also processed into fish powder for inclusion in infant formulas, which has occasionally triggered allergic responses. The purpose of this study was to examine how glycosylation and UHP treatment affect the structure and immunobinding activity of PV in Salangid icefish.

## 2. Material and Methods

### 2.1. Materials

Fresh-frozen Salangid icefish was purchased from Suzhou Yuannong Food Co., Ltd. (Suzhou, China), which are not alive. Mouse anti-mackerel PV monoclonal antibody, horseradish peroxidase (HRP)-conjugated goat anti-mouse IgG antibody, and mackerel PV antigen were purchased from Shenzhen Anti Biological Technology Co., Ltd. (Shenzhen, China). The enhanced chemiluminescence kit utilized for Western blotting was purchased from Beyotime Biotech Inc. (Shanghai, China). Non-fat milk, Tween 20, nitrocellulose membranes, and 15% precast gels for Tris-Glycine PAGE with 10 wells were purchased from Sangon Biotech Co., Ltd. (Shanghai, China).

Our study did not require further ethics committee approval as it did not involve animal or human clinical trials and was not unethical.

### 2.2. Extraction of PV

The extraction procedure was based on the method described by Dasanayaka et al. [24]. The 100 g of Salangid icefish was homogenized with 300 mL of 20 mM pH 7.4 Tris-HCl buffer by a food blender (Hangzhou Jiuyang Living Appliance Co., Ltd., Hangzhou, China) and then centrifuged (Neofuge 15R high speed refrigerated centrifuge, Shanghai Lishen Scientific Equipment Co., Ltd., Shanghai, China) at 8500 rpm for 20 min at 4 °C. After centrifugation, the supernatant was subjected to heat treatment at 100 °C for 15 min, followed by centrifugation at 8500 rpm and 4 °C for 15 min. The supernatant after heat treatment was referred to as the heat extract. Solid ammonium sulfate was added to the heat extract to a saturation of 60%. After resting at 4 °C for 2 h, the supernatant was then treated with solid ammonium sulfate to achieve a saturation level of 80%. After another 2 h of resting at 4 °C, solid ammonium sulfate was added to the supernatant post centrifugation to reach 100% saturation, followed by a 2 h rest at 4 °C and centrifugation at 8500 rpm and 4 °C for 25 min. The precipitates collected after centrifugation were dissolved with distilled water, and the distilled water was used as a dialysis buffer for dialysis at 4 °C. Finally, the protein concentration was determined using the Bradford reagent kit.

### 2.3. Glycation

Five reducing sugars (ribose, xylose, glucose, fructose, and galactose) were selected for glycation, and 5 mL of 3 mg/mL PV solution was reacted with reducing sugars at a ratio of 1:10 (*w/w*) in a boiling water bath for 30 min.

### 2.4. UHP Treatment

The 3 mg/mL PV solution was treated at 20 °C for 20 min under pressure of 200 MPa, 300 MPa, 400 MPa, 500 MPa, and 600 MPa, respectively. The UHP treatment was conducted in a Shanghai Litu Ultra High Voltage Equipment Co., Ltd. (Shanghai, China), and the equipment model is FB-110G.

### 2.5. SDS-PAGE Analysis

The 20 μL sample was mixed with 5 μL loading buffer and heated at 100 °C for 5 min. Subsequently, these samples were loaded into a 15% precast Tris-Glycine PAGE gel and electrophoresed at a constant voltage of 150 V for 1 h. Then the gel was stained with Coomassie brilliant blue staining solution and decolorized by decolorizing solution. The molecular weight of the PV in each sample was determined by a pre-dyed protein marker as a standard.

### 2.6. Western Blot Analysis

Western blot analysis was performed as described by De Jongh et al. [25]. Briefly, PV samples from the polyacrylamide gel were transferred to a 0.2 μm nitrocellulose membrane (NC), followed by blocking with 5% skimmed milk in TBST (20 mM Tris-HCl, pH 7.4, containing 0.145 M NaCl and 0.05% Tween 20) for 2 h at room temperature. The membrane was then incubated with the mouse anti-fish PV monoclonal antibody for another 2 h at room temperature. After washing with TBST, the membrane was incubated with HRP-conjugated goat anti-mouse IgG for 1 h. Antibody binding was detected using enhanced chemiluminescence (ECL).

### 2.7. Identification by Mass Spectrometry (MS)

Target protein bands were excised from the SDS-PAGE gel and reduced with dithiothreitol (90 μL of 100 mM NH_4_HCO_3_ and 10 μL of 100 mM DTT for 30 min at 5 °C). They were then alkylated with iodoacetamide (70 μL of 100 mM NH_4_HCO_3_ and 30 μL of 100 mM IAA in the dark for 20 min at room temperature) and digested with trypsin (25 mM NH_4_HCO_3_, 5 μL) at 37 °C for 20 h. A volume of 0.7 μL of the digested sample was spotted onto the target plate and overlaid with 0.7 μL of matrix solution. The samples were processed by MALDI-TOF/TOF MS (Bruker Corporation, Karlsruhe, Baden-Württemberg, Germany) and searched by Mascot software (Mascot Server 2.8.3, Matrix Science Ltd., London, UK) [26]. The search type was MS/MS Ion Search.

### 2.8. Analysis of the Glycation PV’s Browning Intensity

During the MRs, colorless compounds with strong UV-absorbing properties are generated in the intermediate stages, while brown polymers form in the final stages [27]. Therefore, the degree of MR was analyzed by monitoring absorbance at specific wavelengths. The PV concentration was diluted to 1 mg/mL and loaded into a 96-well plate. The absorbance at both 420 nm and 294 nm was measured by a multimode microplate reader (SpectraMax M2, Molecular Devices, Shanghai, China) [28].

### 2.9. Analysis of the Glycation Degree of PV

The method of Vissers et al. [29] was adapted to analyze free amino groups using the o-phthaldialdehyde (OPA) method to evaluate the glycation degree of PV. The OPA reagent was prepared by combining 17 mg of dithiothreitol (DTT), 0.762 g of sodium tetraborate, 20 mg of sodium dodecyl sulfate (SDS), and 4 mg of OPA, dissolved in 0.4 mL of ethanol, with the final volume brought to 20 mL with distilled water. Twenty μL PV (1 mg/mL) was mixed with 200 μL of the OPA reagent and incubated at 37 °C for 2 min. The absorbance was measured at 340 nm by a multimode microplate reader. The available amino groups were estimated using a calibration curve established with L-leucine as the standard.

### 2.10. Ultraviolet (UV) Absorption Spectroscopy Analysis

UV absorbance spectra were recorded using a multimode microplate reader (SpectraMax M2, Molecular Devices, USA) with a 96-well plate at a PV concentration of 1 mg/mL. The spectra were scanned from 250 nm to 450 nm [30]. UV absorbance spectra were plotted using Origin Software 2024 (OriginLab Corporation, Northampton, MA, USA).

### 2.11. CD Spectroscopy Analysis

CD spectroscopy was obtained with a Chirascan spectrometer (Applied Photophysics Limited, Leatherhead, Surrey, UK). Each sample was diluted to 0.2 mg/mL with double-distilled water and measured using a 1 mm quartz cuvette. The samples were scanned three times with the wavelength range of 190 to 260 nm at 1 nm/s [31]. The secondary structure content was determined using the CDNN program (version 2.0).

### 2.12. Fluorescence Spectroscopy Analysis

Fluorescence properties of MR products and intrinsic fluorescence of PV were analyzed using a fluorescence spectrophotometer (FLS980; Edinburgh Instruments Ltd., Livingston, Scotland, UK). The PV concentration was 1 mg/mL, with an excitation wavelength of 295 nm for intrinsic fluorescence and emission spectra recorded from 300 nm to 500 nm. Additionally, the fluorescence properties of glycation PVs were measured at λex = 347 nm and λem = 400–600 nm [32].

### 2.13. Surface Hydrophobicity

Surface hydrophobicity of glycation PVs was analyzed according to Zhao et al. [33]. The protein concentration was adjusted to 0.5 mg/mL; 80 µL of 8 mM ANS was added to a 2 mL sample, which was then incubated for 10 min before measuring fluorescence intensity at λex = 370 nm and λem = 400–600 nm.

### 2.14. Statistical Analysis

All data were presented as mean ± standard deviation (SD) of at least three independent experiments. One-way analysis of variance (ANOVA) was applied to analyze the effects of glycation and ultra-high pressure on the variables measured. Duncan’s multiple comparisons was used to assess the statistical significance of the means using IBM SPSS Statistics 20.0 (SPSS Inc., Chicago, IL, USA).

## 3. Results and Discussion

### 3.1. Analysis of Protein Composition During Extraction

**Figure 1:**
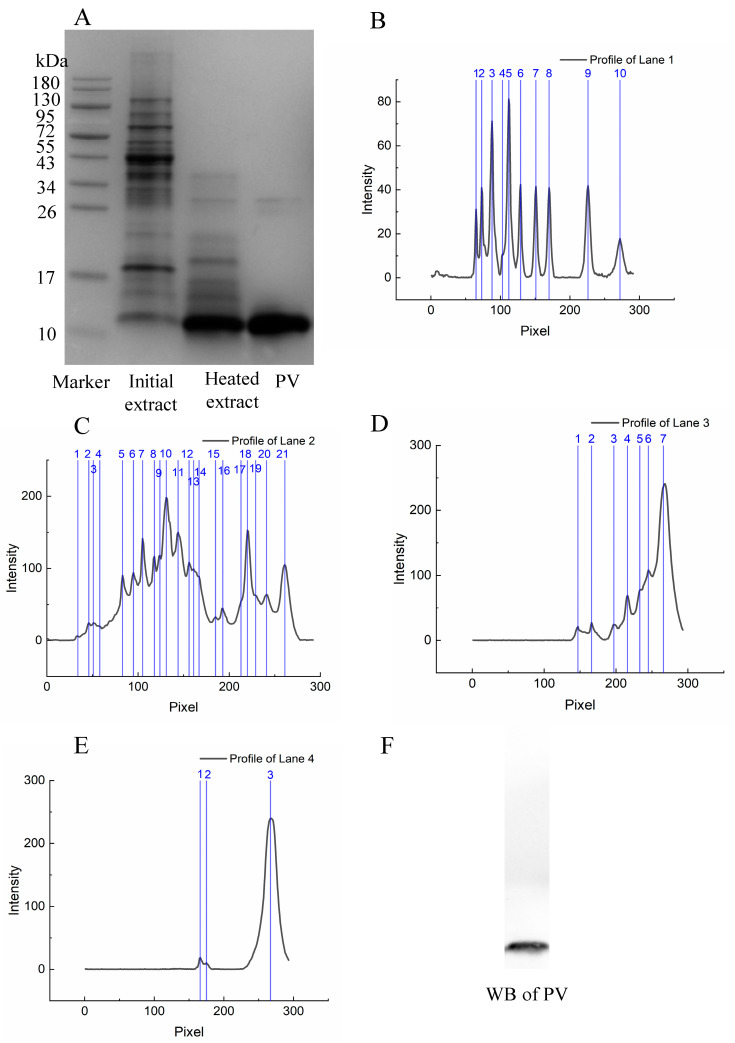
SDS-PAGE, electrophoretic band intensity (The numbers in blue are the band in the lane) and Western blotting analysis of PV in Salangid icefish during extraction. (**A**) SDS-PAGE of PV; (**B**) Band intensity of lane 1; (**C**) Band intensity of lane 2; (**D**) Band intensity of lane 3; (**E**) Band intensity of lane 4; (**F**) Western blotting of PV.

In this study, SDS-PAGE was employed to evaluate alterations in the protein composition during the extraction of Salangidae icefish (Figure 1A). Figure 1C–E shows the strength of the electrophoretic bands for Salangid icefish PV during extraction. Lanes 1–4 correspond to lanes in Figure 1B–E. It can also be seen in Figure 1B–E that the types and content of miscellaneous proteins also decrease during the extraction process. In addition, the molecular weight and mobility of the bands are analyzed in Table S1.

The electrophoresis results indicated a significant amount of impurities in the crude extract. After boiling, impurities above 35 kDa were removed, followed by ammonium sulfate precipitation to remove unwanted proteins below 35 kDa. The gray value of lane 4 was calculated using imageJ to indicate the purity of PV. Ultimately, the purity of the target protein obtained exceeded 90% (Table S2).

### 3.2. Identification of PV

**Figure 2:**
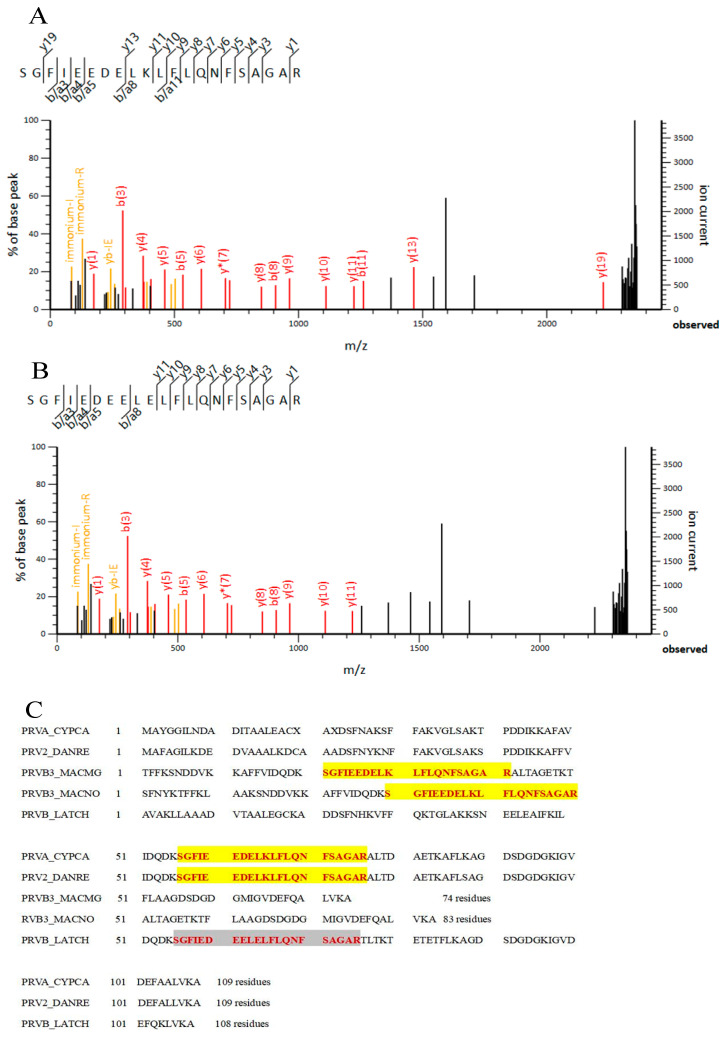
Identification by MSof Salangid icefish PV. (**A**) MS/MS Fragmentation of SGFIEEDELKLFLQNFSAGAR; (**B**) MS/MS Fragmentation of SGFIEDEELELFLQNFSAGAR; (**C**) Results of sequence match, with the matched peptides displayed in red font, while the yellow and gray highlights indicate different peptide sequences.

A clear band near 10 kDa is visible on the SDS-PAGE gel (Figure 1A). Western blotting analysis confirmed that this band corresponds to PV (Figure 1F). Shruti et al. [17] showed that immunoblotting of fish raw extracts by monoclonal antibodies indicated PV molecular weights from different fish species between 10 and 15 kDa, which is in agreement with the results of this study. To further identify the protein, MALDI-TOF/TOF mass spectrometry was used to analyze the excised PV band from the SDS-PAGE gel. Individual ion scores above 41 indicate identity or extensive homology (*p* < 0.05). The scores for PV alpha (*Cyprinus carpio*), PV-2 (*Danio rerio*), and PV beta 3 (fragments of *Macruronus magellanicus* and *Macruronus novaezelandiae*) were all 89, while PV beta (*Latimeria chalumnae*) scored 62, all exceeding the cutoff for individual ions. The matched fragment ion spectrum is shown in Figure 2A,B.

The proteins and peptides successfully matched are displayed in Figure 2C, with matched peptides highlighted in red and different peptide sequences in yellow and gray. The matched peptide fragment had a molecular weight of 2.371 kDa, and its secondary mass spectrum matched the database, confirming the protein as PV.

### 3.3. Analysis of Glycation PV Characteristics

**Figure 3:**
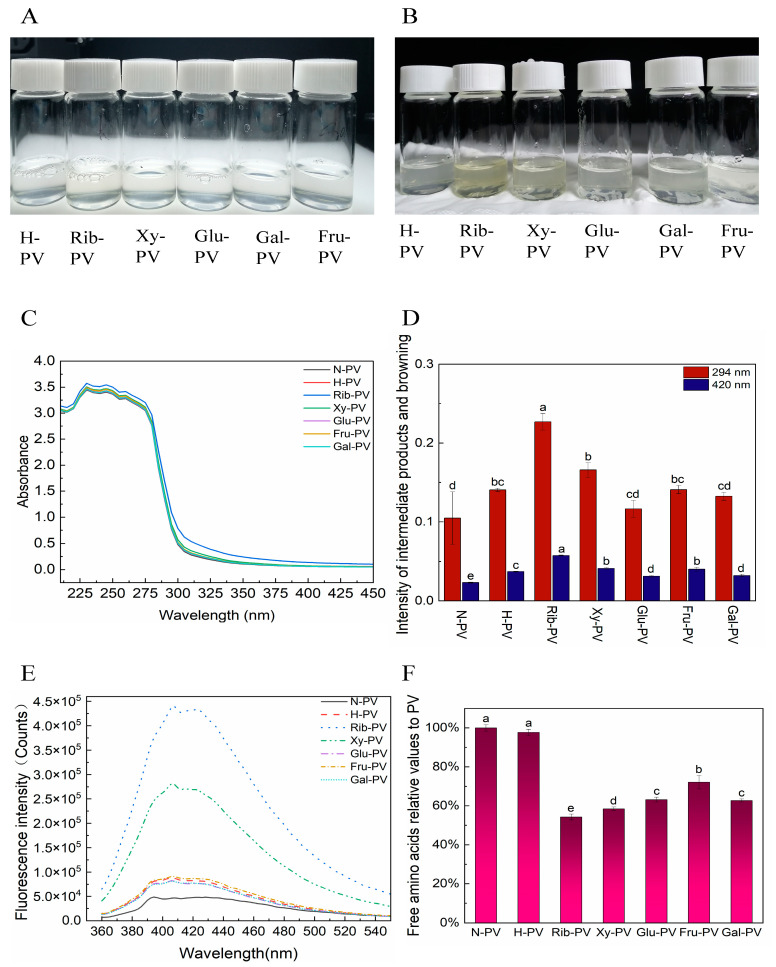
Analysis of glycation degree of PVs. (**A**,**B**): Color changes of PV solutions before and after glycation with different reducing sugars; (**C**) UV absorption spectrum of glycated PV; (**D**) Comparison of UV absorbance characteristics of PV samples at 294 nm and 420 nm (Differences in letters indicate significant differences); (**E**) Fluorescence properties of PV samples (ex = 347 nm); (**F**). Degree of PV glycation. (N-PV: Untreated PV; H-PV: heated PV; Rib-PV: PV reacting with ribose; Xy-PV: PV reacting with xylose; Glu-PV: PV reacting with glucose; Gal-PV: PV reacting with galactose; Fru-PV: PV reacting with fructose).

Figure 3A,B illustrate the color evolution in solutions containing ribose and xylose as reaction substrates during glycation, with the most significant changes observed in the presence of ribose.

Glycation can alter the UV absorption spectral properties of proteins [34]. Thus, to investigate structural modifications from the reaction of reducing sugars with PV, the UV absorption spectra of PV glycation products with five reducing sugars were examined in the 250–450 nm range. Since phenylalanine (Phe), tyrosine (Tyr), and tryptophan (Trp) have conjugated double bonds, there is absorption in the UV region. Whereas, previous studies have shown that PV contains high levels of Tyr and Phe and is deficient in Trp [35,36,37]. This results in a characteristic absorption peak at 260 nm when the PV is scanned in the UV [38]. As shown in Figure 3C, PV exhibits UV absorption peaks at 230 nm, 245 nm, 260 nm, and 275 nm, while Phe peaks at 222 nm and 259 nm, and Tyr peaks at 231 nm and 272 nm. Thus, this result also confirmed the presence of Phe and Tyr in the PV. After glycation, the intensity of UV absorption peaks increased compared to natural PV, although the change was subtle. This is inconsistent with the results of previous studies. Wu et al. [34] showed that the addition of glucose for glycation significantly reduced the UV absorption of PV, while Zhang et al. [39] noted increased UV absorption after glycation. The discrepancies may stem from variations in the types and concentrations of reducing sugars, the ratios of reducing sugars to PV, and the glycation conditions employed.

During the intermediate stages of MRs, a carbonyl group condenses with an amino group to form colorless compounds. These compounds exhibit a characteristic absorption peak at 294 nm [27,40,41]. Browning intensity, indicated by absorbance at 420 nm, fluctuated with the presence of reducing sugars (Figure 3D). Spectrophotometric analysis revealed that solutions with ribose, xylose, and fructose as substrates exhibited higher absorbance at both 294 nm and 420 nm, with ribose showing the highest values. These findings suggest that ribose-treated PV produced more intermediate products and increased browning intensity. Additionally, the type of reducing sugar has a significant effect on the degree of browning, which is consistent with the results of previous studies [42,43].

FS is frequently utilized to research MRs. Numerous studies have shown its effectiveness in characterizing variations in MR products within complex food systems [44]. An absorption peak was detected at 400–425 nm (406 and 422 nm) (Figure 3E), consistent with the fluorescence intensity of glycation products. This suggests that the peak is due to glycation. As the MR progressed, fluorescent compounds initially accumulated and were later rapidly consumed, while browning products increased [45]. Strong fluorescence was detected in this wavelength range for R-PV and X-PV, with R-PV exhibiting higher intensity. This indicates that ribose treatment produced more fluorescent products.

The free amino groups on protein side chains undergo dehydration condensation with carbonyl groups of reducing sugars, transitioning to a bound state. The quantity of these bound amino groups indicates the degree of glycation [46]. A higher binding degree reflects an increased content of bound amino groups, suggesting enhanced glycation. As shown in Figure 3F, treatment with five different reducing sugars decreased the free amino content of PV, indicating that all can glycate PV. Among them, ribose and xylose demonstrated greater binding to the free amino groups of PV, reaching 58.42% and 54.26%, respectively, suggesting they have the highest glycation potential. Previous studies have shown that the degree of reduction in free amino content after the MR is related to the type of reducing sugar. This is consistent with the findings of this research [30,31,34,39]. In addition, Zhang et al. [39] showed that after the MR, the free amino acid content was in the order of fructose > glucose > galactose > ribose in descending order. It is basically consistent with the results of the present study, in which fructose has the lowest degree of glycation, possibly because aldehydes are more reactive than ketoses.

### 3.4. Analysis of CD Spectroscopy

**Figure 4:**
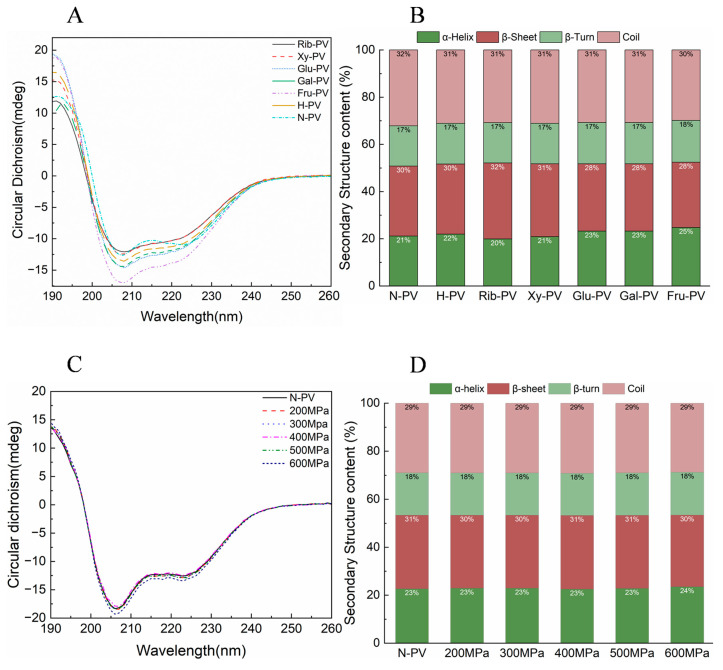
CD spectrum of PVs after glycation and UHP treatment. (**A**) CD spectra of PV before and after glycation; (**B**) Proportion of secondary structure composition in PVs after glycation; (**C**) CD spectra of PV before and after UHP; (**D**) Proportion of secondary structure composition in PVs after UHP treatment. (N-PV: Untreated PV; H-PV: heated PV; Rib-PV: PV reacting with ribose; Xy-PV: PV reacting with xylose; Glu-PV: PV reacting with glucose; Gal-PV: PV reacting with galactose; Fru-PV: PV reacting with fructose.).

CD was used to analyze changes in the secondary structure of PV (proteins or peptides) before and after glycation. The data were processed using CDNN software to determine the percentages of α-helix, β-sheet, β-turn, and random coil structures. The spectrum revealed three characteristic peaks: two negative peaks at 209 and 222 nm, and a positive peak at 190 nm, corresponding to α-helical and β-folded structures, respectively (Figure 4A). Previous studies measured the CD spectrum of PV treated at different temperatures for 2 h. They found three peaks at 198, 208, and 222 nm, which are similar to the results of this study [47].

Compared to PV before glycation, the secondary structure displayed significant alterations depending on the type of reducing sugar used (Figure 4B). Specifically, ribose-PV showed a decrease in α-helix content and an increase in β-sheet content. In contrast, glucose-PV, galactose-PV, and fructose-PV exhibited increased α-helix content and significant reductions in β-sheet content. The β-turn content remained relatively unchanged across different reducing sugar treatments. Fu et al. [31] also showed that ribose causes a decrease in helix content and a decrease in sheet content.

Compared to untreated PV samples, only the PV treated at 600 MPa showed a slight change in CD (Figure 4C). The other UHP-treated samples exhibited no significant changes in intensity or peak positions. (Figure 4D). This suggested that UHP treatment has a negligible impact on the secondary structure of PV.

The CD studies indicated that different reducing sugars can induce changes in the secondary structure of PV, whereas UHP treatment has a limited effect. Wang et al. [48] summarized that significant alterations in protein secondary structure typically require relatively high pressure (>400 MPa), as lower pressures result in minimal or no changes. This study further confirms that lower pressures do not affect the secondary structure, which is primarily stabilized by highly incompressible hydrogen bonds.

### 3.5. Intrinsic Fluorescence Properties of PV

Intrinsic fluorescence is a valuable tool for investigating protein tertiary structure. It provides insights into the molecular environment surrounding the chromophores within proteins. Trp and Tyr serve as the primary fluorophores responsible for intrinsic fluorescence emission. Variations in the effects of glycation modifications by different reducing sugars on intrinsic Trp fluorescence characteristics have been reported [49].

**Figure 5:**
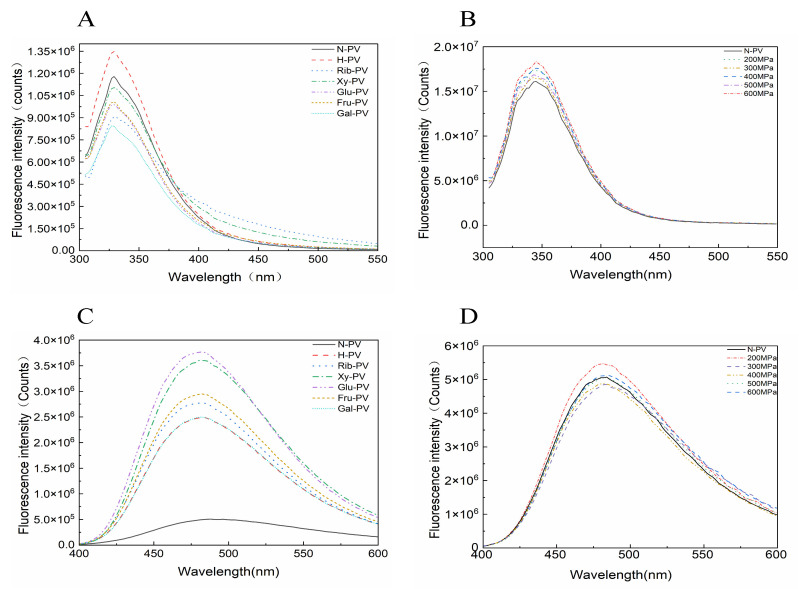
Intrinsic fluorescence and surface hydrophobicity of PV before and after the glycation and UHP treatment. (**A**) Intrinsic fluorescence of PVs after glycation; (**B**) Intrinsic fluorescence of PVs after UHP treatment; (**C**) Surface hydrophobicity of PVs after glycation; (**D**) Surface hydrophobicity of PV after UHP treatment.

The maximum fluorescence absorption peak of PV occurred at approximately 325 nm (Figure 5A), consistent with the characteristics of Trp. However, as glycation progressed, the fluorescence intensity decreased. This was likely due to the modification of Trp residues that became buried within the molecular structure. Among the samples, Gal-PV exhibited the lowest fluorescence absorption, followed by Rib-PV. Zhang et al. [39] reported the fluorescence intensity order as PV > Glu-PV > H-PV > Lac-PV > Rib-PV > Fru-PV, which differs from the present study. This discrepancy may be attributed to variations in buffers and pH for dissolving PV and reaction conditions because ionic strength and pH also cause changes in the microenvironment of amino acid residues in proteins.

Following UHP treatment, the intrinsic fluorescence properties of PV changed, showing a progressive increase in fluorescence intensity with applied pressure in the order of 600 MPa > 400 MPa > 200 MPa > 500 MPa > 300 MPa (Figure 5B). This increase can be attributed to the partial or complete unfolding of PV’s tertiary structure under UHP, which exposes more aromatic amino acid residues and enhances fluorescence intensity. Liu et al. [50] found that steamed and steamed + reverse-pressure sterilized shrimp had increased excitation peaks, stronger fluorescence intensity, and a red shift compared to raw shrimp. This suggests that pressure treatment alters the microenvironment of Trp residues and reveals their hydrophobic groups. The discrepancy between these results and the present study may be due to PV being a stress-resistant protein.

In summary, the application of reducing sugar glycation and UHP treatment has the potential to alter the tertiary structure of PV.

### 3.6. Analysis of Surface Hydrophobicity

Surface hydrophobicity is crucial for maintaining protein tertiary structure. The ANS fluorescence probe was used to assess the impact of glycation on molecular surface hydrophobicity. A strong correlation between glycation and protein surface hydrophobicity has been reported [33]. As shown in Figure 5C, the hydrophobicity of PV modified by reducing sugars significantly increased compared to control PV, especially around the 480 nm fluorescence peak. This suggests that glycation induced structural changes, exposing hydrophobic groups previously buried within the protein. The fluorescence intensities of H-PV, Rib-PV, Xy-PV, Glu-PV, Fru-PV, and Gal-PV all showed varying degrees of enhancement relative to PV. Notably, glucose significantly increased the hydrophobicity of PV. This is likely because the aldehyde groups added to the modified PV created more steric hindrance, making its hydrophobicity more accessible. Previous studies have shown that glycation modifications increase the hydrophobicity of PVs [33,39], which aligns with our findings.

UHP treatment exerts varying effects on the surface hydrophobicity of PV (Figure 5D). Surface hydrophobicity increased with 200 MPa and 600 MPa treatments, while 500 MPa treatment showed no significant change, as its fluorescence spectrum nearly overlapped with that of untreated PV. In contrast, hydrophobicity decreased at 300 MPa and 400 MPa. These findings suggest that UHP treatment affects PV’s surface hydrophobicity and tertiary structure, but the effects are small. Liu et al. [50] reported a significant increase in surface hydrophobicity in steamed + back-pressure sterilized shrimp compared to raw and steamed shrimp. This discrepancy with our study may arise from differences in treatment conditions and protein species. Additionally, the effects of high pressure on PV are complex [51,52]. They can cause decreased hydrophobicity within a certain pressure range but increase hydrophobicity outside that range.

### 3.7. The Changes of Molecular Weight and Immunobinding Activity of PV

**Figure 6:**
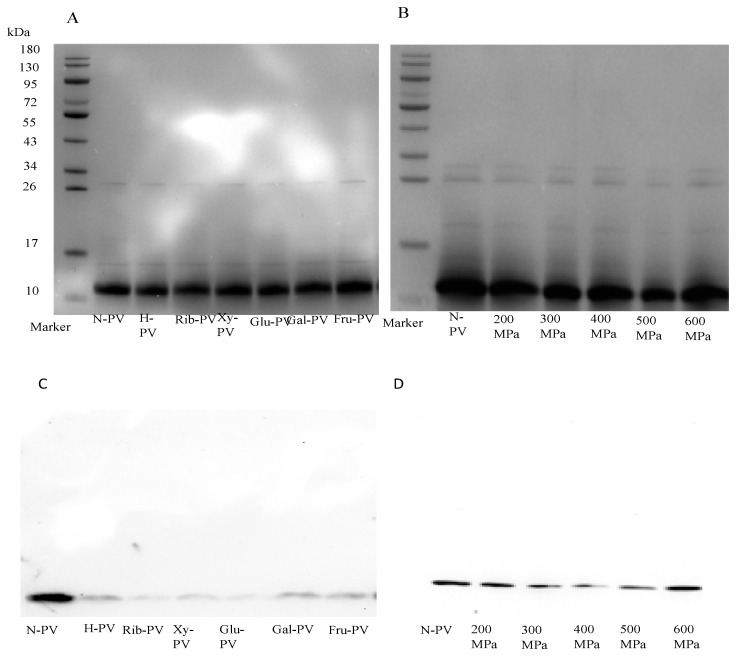
Molecular weights and the IgG immunobinding activities of PVs before and after the glycation and UHP treatment. (**A**) SDS-PAGE of PVs before and after glycation; (**B**) SDS-PAGE of PVs before and after UHP treatment; (**C**) Western blot of PVs before and after glycation; (**D**) Western blot of PVs before and after UHP treatment. (N-PV: Untreated PV; H-PV: heated PV; Rib-PV: PV reacting with ribose; Xy-PV: PV reacting with xylose; Glu-PV: PV reacting with glucose; Gal-PV: PV reacting with galactose; Fru-PV: PV reacting with fructose.).

Electrophoresis results (Figure 6A,B) indicated that glycation and high-pressure treatment did not significantly affect the molecular weight of PV. This contradicts the findings of Zhao et al. [33], who reported that the MR resulted in the formation of dimers and an increase in dispersion bands. This discrepancy may be attributed to differences in the time and temperature conditions of the MR. Zhao et al. [33] mixed the sample with rPV: glucose ratio of 1:3 (wt/wt), and after freeze-drying, reacted at 60 °C for 72 h. Therefore, the formation of dimers may be caused by too long of a reaction time.

Alterations in the conformation of PV may impact its immunobinding activity. In Western blot analysis, lower grayscale values indicate reduced IgG immunobinding activity. As shown in Figure 6C, glycation decreased the immunobinding activity of PV, with different reducing sugars exhibiting varying effects. PV glycated with glucose showed the lowest grayscale value, followed by those modified with ribose and xylose, while fructose and galactose yielded similar values (Table S5). The reduced IgG immunobinding activity due to glycation may result from the longer carbon chains of hexose molecules and the presence of aldehyde groups, which increase steric hindrance and affect the conformation of PV. This is further supported by the observed changes in surface hydrophobicity. Consequently, glycation can decrease the IgG immunobinding activity of PV, presumably reducing its allergenicity.

Previous studies have indicated that glycation reduces the immunoreactive activity of PV. However, Zhang et al. [39] reported that the IgG/IgE binding capacity of Lac-PV, Rib-PV, and Gal-PV was lower, while Glu-PV and Fru-PV exhibited higher binding capacity. These findings are inconsistent with the present study and may be attributed to differences in reaction conditions. Zhang et al. [39] mixed reducing sugar and PV at a ratio of 1:1; it was lyophilized and finally incubated at 60 °C and 65% relative humidity for 1 h. Additionally, variations in the type and concentration of IgG may also influence the results.

Figure 6D demonstrates that UHP treatment affects the IgG immunobinding activity of PV, with varying impacts depending on the treatment pressure. Specifically, PV treated at 400 MPa, 300 MPa, and 500 MPa exhibited decreased IgG immunobinding activity. But the treatment at 200 MPa and 600 MPa resulted in no significant changes (Table S6). These results indicate that UHP treatment influences IgG immunobinding activity in an irregular manner. This irregularity may arise from different pressures inducing distinct conformational changes, which subsequently affect immunobinding activity, as also reflected in changes in surface hydrophobicity. Yang et al. [53] reported no effect of high-pressure treatment on immunobinding active substances. The discrepancies observed may be attributed to differences in the temperature and pressure conditions of the treatments employed. Jia et al. [54] investigated the IgG/IgE binding ability of β-lactoglobulin. They found that IgG binding increased with pressure and was higher than the control group, surpassing the control group. In contrast, IgE binding affinity decreased between 100 and 200 MPa, reaching its lowest point at 200 MPa, before rising again between 200 MPa and 300 MPa. Judit et al. [55] summarized the effects of high-pressure treatment on PV sensitization. They noted that pressure below 300 MPa had little effect on sensitization, whereas higher pressures and temperatures are necessary for a significant reduction. These findings indicate that the effect of UHP on PV immunoreactivity is complex and may be influenced by factors such as ionic strength, pH, pressure, and temperature, highlighting PV’s sensitivity to these conditions.

## 4. Conclusions

This study extracted and identified PV from Salangid icefish. We investigated the impact of glycation and UHP on the structure and IgG immunobinding activity of PV. The results indicated that glycation with ribose or xylose significantly affected the structure of PV. Glycation altered both the secondary and tertiary structures of the PV protein, thereby influencing its IgG immunobinding activity. In addition, the results from UHP treatment showed that different pressures had varying effects on the structure of PV and its IgG immunobinding activity, but these effects were not pronounced. In the future, we will study how the MR time and temperature affect the structure of PV and its immunobinding capacity. This research provides a potential basis for the safety of Salangid icefish processed products.

## Data Availability

The original contributions presented in this study are included in the article/Supplementary Material. Further inquiries can be directed to the corresponding author.

## Supplementary Materials

The following supporting information can be downloaded at: https://www.mdpi.com/article/10.3390/foods14050856/s1, Table S1. The Grayscale values and content of the target protein on SDS-PAGE (Figure 1A). Table S2. Electrophoretic bands and molecular weight analysis of PV extraction from Salangid Icefish. Table S3. The information of matched Peptide. Figure S1. Mass spectra of purified salangid icefish parvalbumin. Table S4. Summary of MALDL-TOF/TOF mass spectrometry. Figure S2. Standard curve for the determination of free amino acid content utilizing L-leucine as the calibration standard. Table S5. Grayscale values of the WB plot after the Maillard reaction with different Reducing sugar. Table S6. Grayscale values of the WB plot after the ultrahigh pressure processing with different pressure.
