# Supplementary material for: A Comparison of the Structural Changes and IgG Immunobinding Activity of Parvalbumin in Salangid Icefish (Neosalanx taihuensis) After Glycation and Ultra-High Pressure Treatment
Source: Foods. 2025 Mar 2;14(5):856. doi: 10.3390/foods14050856 (PMC11898409; doi:10.3390/foods14050856)
Supplement: Supplementary file 1 [file foods-14-00856-s001.zip › foods-3473681-supplementary.pdf]

Supplementary data

A Comparison of the Structural Changes and IgG Immunobinding Activity of Parvalbumin in Salangid Icefish (Neosalanx taihuensis) After Glycation and Ultra-High Pressure Treatment

Ying Huang, Yang Hu, Jiawei Liu and Haiying Liu *

School of Food Science and Technology, Jiangnan University (214122), Wuxi, China;
6220111044@stu.jiangnan.edu.cn (Y.H.); 6230112026@stu.jiangnan.edu.cn (Y.H.); 6210112050@stu.jiangnan.edu.cn (J.L.)

* Correspondence: liuhaiying@jiangnan.edu.cn; Tel./Fax: +86-510-85329076

Table S1. The Grayscale values and content of the target protein on SDS-PAGE(Fig. 1A).

Lane	Band	Relative Migration	Molecular Weight （kDa）	Lane	Band	Relative Migration	Molecular Weight (kDa)
Lane2	Band 1	0.11644	534.29956	Lane2	Band 17	0.72945	18.84526
Lane2	Band 2	0.15753	333.22351	Lane2	Band 18	0.75342	17.7651
Lane2	Band 3	0.17466	278.08788	Lane2	Band 19	0.78425	16.41473
Lane2	Band 4	0.19863	219.16025	Lane2	Band 20	0.82534	14.65148
Lane2	Band 5	0.28425	106.7046	Lane2	Band 21	0.89384	11.74864
Lane2	Band 6	0.32534	80.60544	Lane3	Band 1	0.50342	34.59526
Lane2	Band 7	0.35959	65.61826	Lane3	Band 2	0.56849	28.2303
Lane2	Band 8	0.40411	51.96188	Lane3	Band 3	0.67466	21.49476
Lane2	Band 9	0.42466	47.20413	Lane3	Band 4	0.73973	18.37787
Lane2	Band 10	0.44863	42.55765	Lane3	Band 5	0.79795	15.82364
Lane2	Band 11	0.49315	35.8629	Lane3	Band 6	0.83904	14.06826
Lane2	Band 12	0.53425	31.26482	Lane3	Band 7	0.91096	11.02984
Lane2	Band 13	0.55137	29.67262	Lane4	Band 1	0.56655	28.38694
Lane2	Band 14	0.57192	27.95734	Lane4	Band 2	0.59727	26.08368
Lane2	Band 15	0.63356	23.76104	Lane4	Band 3	0.91126	11.01729
Lane2	Band 16	0.66096	22.21618				

Table S2. Electrophoretic bands and molecular weight analysis of PV extraction from Salangid Icefish.

lane3	1	2	3	sum	purity
Grayscale values	538.506	343.849	18615.773	19498.128	0.955

Table S3. The information of matched Peptide.

Query	Observed	Mr(expt)	Mr(calc)	ppm	M	Score	Expect	Rank	Unique	Peptide
5	2371.5329	2370.5256	2370.1855	143	1	89	1.10E-06	1	U	K.SGFIEEDELKLFLQNFSAGAR.A
	2371.5329	2370.5256	2371.1332	-256.22	0	62	0.00052	2	U	K.SGFIEDEELELFLQNFSAGAR.T

Figure S1. Mass spectra of purified salangid icefish parvalbumin.

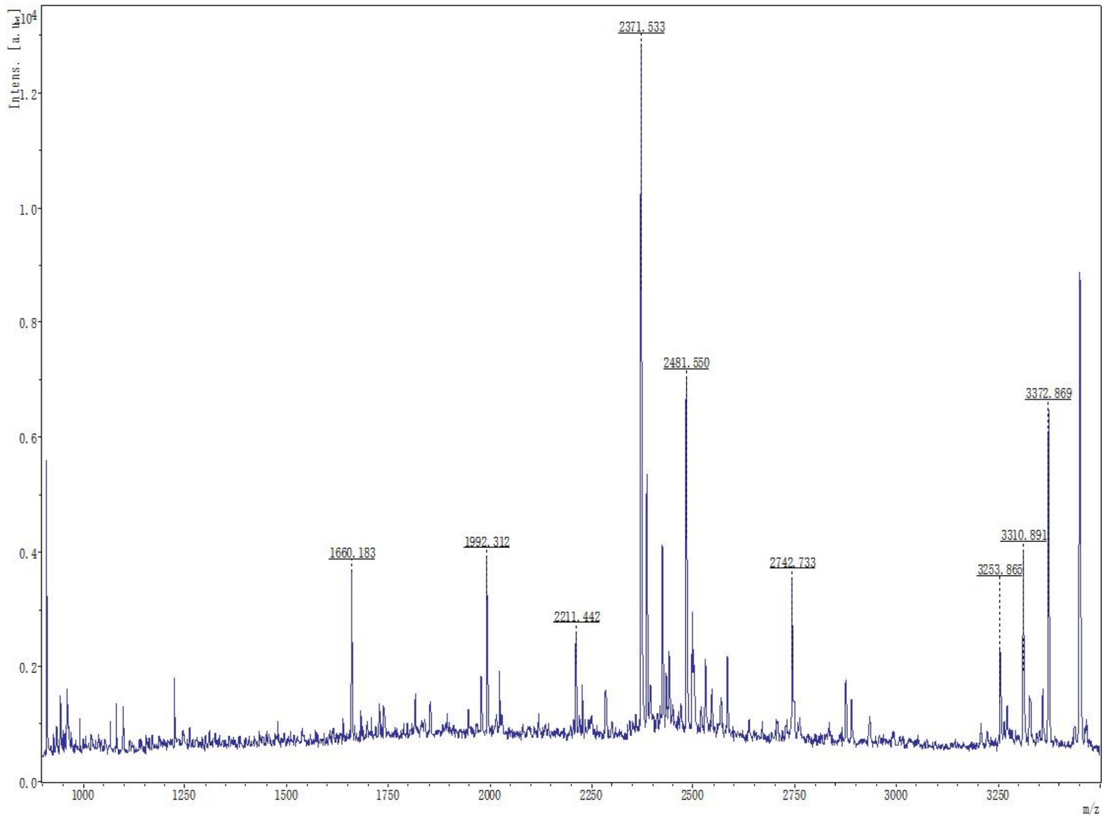

Table S4. Summary of MALDL-TOF/TOF mass spectrometry.

Start–End	Accession no.	Score	Mass	Matches	Sequences	Calculated pI	Protein sequence coverage	Protein description
56-76	PRVA_CYPCA	89	11444	1 (1)	1 (1)	4.43	0.19	Parvalbumin alpha OS=Cyprinus carpio OX=7962 PE=1 SV=2
56-76	PRV2_DANRE	89	11615	1(1)	1(1)	4.46	0.19	Parvalbumin-2 OS=Danio rerio OX=7955 GN=pvalb2 PE=3 SV=3
21-41	PRVB3_MACMG	89	8023	1(1)	1(1)	4.34	0.28	Parvalbumin beta 3 (Fragments) OS=Macruronus magellanicus OX=92050 PE=1 SV=1
30-50	PRVB3_MACNO	89	9046	1(1)	1(1)	4.62	0.25	Parvalbumin beta 3 (Fragments)OS= Macruronus novaezelandiae OX=248764 PE=1 SV=1
55–75	PRVB_LATCH	62	11725	1 (1)	1 (1)	4.68	0.19	Parvalbumin beta OS=Latimeria chalumnae OX=7897 PE=1 SV=1

Figure S2. Standard curve for the determination of free amino acid content utilizing L-leucine as the calibration standard.

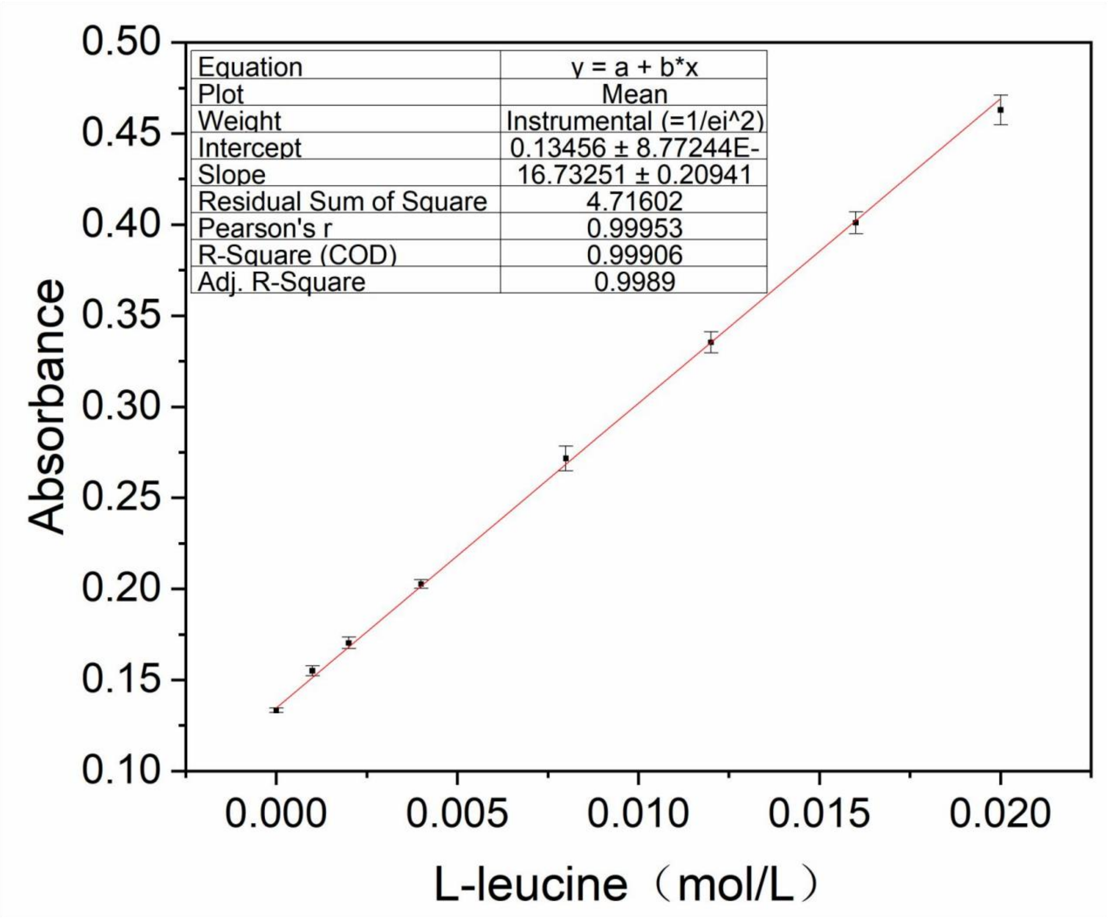

Table S5. Grayscale values of the WB plot after the Maillard reaction with different Reducing sugar.

	N-PV	H-PV	Rib-PV	Xy-PV	Glu-PV	Gal-PV	Fru-PV
Grayscale values	23781.368	3883.861	698.163	1194.77	433.092	3443.054	3347.74

Table S6. Grayscale values of the WB plot after the ultrahigh pressure processing with different pressure.

sample	N-PV	200MPa	300MPa	400MPa	500MPa	600MPa
Grayscale values	13745.447	11446.083	8310.569	5064.619	7028.276	13209.012
rate	1.000	0.833	0.605	0.368	0.511	0.961
